# GBA/GBN-position on the feedback of incidental findings in biobank-based research: consensus-based workflow for hospital-based biobanks

**DOI:** 10.1038/s41431-023-01299-8

**Published:** 2023-02-03

**Authors:** Joerg Geiger, Joerg Fuchs, Madeline Starke, Michael Neumann, Ronny Baber, Sara Y. Nussbeck, Michael Kiehntopf, Cornelia Specht, Thomas Illig, Michael Hummel, Roland Jahns

**Affiliations:** 1 Interdisciplinary Bank of Biomaterials and Data Wuerzburg (ibdw), University and University Hospital Wuerzburg, Wuerzburg, Germany; 2 Leipzig Medical Biobank, University Leipzig, Leipzig, Germany; 3 University Medical Center Goettingen, Central Biobank, UMG, Goettingen, Germany; 4 Institute of Clinical Chemistry and Laboratory Diagnostics and Integrated Biobank Jena (IBBJ), Jena University Hospital, Jena, Germany; 5 German Biobank Node, Charité - Universitätsmedizin Berlin, Berlin, Germany; 6 Hanover Unified Biobank (HUB), Hanover, Germany

**Keywords:** Ethics, Translational research

## Abstract

Incidental research findings pose a considerable challenge to hospital-based research biobanks since they are acting as intermediaries between healthcare and research. In a joint action the centralized biobank ibdw (Interdisciplinary Bank of Biomaterials and Data Wuerzburg) together with local authorities drafted a coherent concept to manage incidental research findings in full compliance with relevant ethical and data privacy regulations. The concept was developed and elaborated in close collaboration with the German Biobank Alliance (GBA). Comprehensive documentation of all steps guarantees the traceability of the process. By a mandatory assessment of the findings prior to re-identification of the individual concerned, unnecessary measures can be avoided. The individual’s “right not to know” is respected according to the stipulations of the informed consent. As a general principle any communication with the individual occurs exclusively through the hospital and by competent physicians with appropriate knowledge and communication skills. We propose this scheme as a blueprint for reporting workflows for incidental research findings at hospital-based biobanks.

## Introduction

During the course of clinical diagnostics or medical research, yet undiagnosed patho-physiological conditions, increased risk or predisposition for a disease, or even a manifest disease of an individual can by chance become apparent. For these unintended or unsolicited findings potentially impacting the well-being of the individual, the term “incidental finding” has been coined.

According to commonly accepted ethical standards for human subjects research, incidental findings should be returned to the patient after well balanced consideration of patient autonomy and legal and ethical obligations of the practitioner [1–3]. The criteria for a well-founded medical decision should be the validity and significance of the observation, the actionability of the finding and severity of the (potentially) underlying disease as well as efficacy and tolerability of interventions necessary [4, 5].

Since the availability of analytical methods enabling the generation of highly dimensional data, such as genetic analysis, omics techniques or multiplex assays, the likelihood of unintended findings is no longer limited to clinical diagnosis but has become increasingly relevant in biomedical research [6, 7].

While in the clinical setting and in the context of clinical studies, experienced health professionals are in charge of communicating incidental findings to the patient based on medical standards, there is no equivalent for biobank samples collected under a broad consent and used in biomedical research. Typically, the research is carried out on human biological samples and data obtained from hospital-based (human) research biobanks, uninvolved in clinical care and thus disconnected from the patient or study subject. The clear separation of research from patient care is a necessity to comply with privacy protection regulations. The biobank itself, acting as intermediary between the clinic and the researcher, has no access to identifying data. In consequence the biobank has to assume the thankless task of providing means to relay this information via the treating physician to the respective donor when required by the stipulations in the informed consent, legal or ethical rules.

In the case of sample collections for clinical trials or within the framework of population-based studies the responsibility for the management of the study subjects and hence, conveying incidental findings, rests with the principal investigator [8, 9]. If the biological samples and data have been obtained by or on behalf of the hospital-based biobank, however, the biobank management is in charge of handling incidental findings [10–12]. The task can become particularly challenging, when the respective sample and data have been collected based on a “broad informed consent”, i.e. for no particular study but for future, yet unknown, research projects implying little or no restrictions on the sample-use [13]. Here, at any rate the biobank appears as the sole contact to the researcher, in particular when taking into account the provisioning of samples worldwide and in the long-term future. While for incidental findings gathered in the context of clinical routine or clinical studies there is clear consensus on which and how incidental findings should be returned to the patient, yet for research occurring distal to clinical care, also termed “secondary research”, there are neither definite rules, nor appropriate procedures established to guide the parties involved, such as researchers or biobanks [14].

Ethical principles and legal regulations call for comprehensive protection of donor privacy and the right of self-determination [15]. Adhering to these principles entails dedicated workflows for donor re-contact and communication. The currently preferred procedure for safeguarding donor privacy is based on the entire pseudonymization of personally identifiable information (PII), clinical data, sample data and the actual sample, thus unlinking the information and obscuring the donor’s identity. Inadvertent merging of the data is made nearly impossible by using disjoint storage of the data entities [16]. For this reason, the necessary retracing of a pseudonymized biological sample to the donor may on no account be accomplished directly by the human biobank providing the sample, nor by the researcher employing the respective biological sample.

There is general consensus that incidental research findings may exclusively be communicated in the context of medical treatment and their medical relevance by an experienced physician. Accordingly, the return of incidental findings to a sample donor by the treating physician, clinic or hospital must be organized on the basis of a reliable workflow ensuring compliance with data protection regulations and ethical principles [17].

To achieve this objective, organizational and technical prerequisites must be in place before a sample collection commences. Ethical aspects regarding reporting of potential incidental findings to the sample donor must be considered along with the design of the human biobank or clinical study and the biosample collection strategy [1, 8, 18, 19]. The study protocol must include provisions on methods, workflows and decision processes for the handling of incidental findings. The donor should be informed of the findings, in understandable terms, and comprehensively about risks and benefits and potential consequences of incidental findings. In addition, the consent form must allow for a clear decision on the “right to know” or “right not to know” of incidental findings [8, 18, 20, 21].

## Materials and methods

The ibdw (**I**nterdisciplinary Bank of Biological Materials and Data Wuerzburg) [22] is the centralized biobank of the University of Wuerzburg and the University Hospital Wuerzburg. Together with the Ethics Committee of the University of Wuerzburg, the institutional data protection officer (DPO), and the legal department of the University Hospital Wuerzburg the management board of the ibdw developed a, to some extent, integrated generic concept for the management of incidental research findings, including templates for documentation. The concept is meant to cover any case of incidental research findings derived from human biological samples and the related data. Primarily the concept is intended for large, interdisciplinary human biobanks collecting human biological samples on the basis of a “broad consent” and offering such samples world-wide.

The integrated concept was presented and discussed in several sessions of the steering committee of the German Biobank Alliance (GBA). The valuable comments received were included in the concept after they were carefully considered. The iterative optimization by the GBA Writing Task Force led to a comprehensive and consistent concept, which was ultimately unanimously accepted by all authors. The procedure provides a coordinated approach for passing and communicating incidental finding(s) of a researcher to the patient concerned, thereby keeping any PII strictly confidential. The workflow was designed to be easily adaptable to different legal or ethical requirements.

## Results

The process is composed of a sequence of seven major steps and documentation of each step is mandatory (see Supplementary Fig. 2 for the template of a “notification form” as implemented in the University Hospital of Würzburg).

The initial report of an incidental finding is documented with a paper-based notification form which is used continuously as an accompanying document during processing the reported finding. The form is passed between the parties involved as indicated on the form and routed through the internal mail system as in a circulation procedure (Supplementary Fig. 2). After completion the form is scanned and electronically stored in the data archive of a trusted third party (TTP). The TTP takes a central role in the process since it has oversight and control of the workflow and handles the PII. For samples collected on the basis of a “broad consent” the PII must be strictly kept separate from the pseudonymized sample and sample data to prevent any accidental or intentional re-identification [13, 23, 24]. Pseudonyms and PII should be held by a TTP which can be an officer or an entity having paramount authority in data protection, or a notary’s office [25, 26]. In case the re-identification of a donor becomes necessary the identity of the donor can only be disclosed via the TTP and only to authorized persons or institutions. By completely monitoring the process, the TTP can determine delays and can contact the party responsible for the corresponding step and request to resume the process. The roles and tasks in this workflow are summarized in Table 1.

**Table 1:** Roles and tasks of the parties involved in the workflow for returning incidental findings.

Workflow	Roles					
	Researcher	Biobank	Accredited Pathology, Laboratory Medicine or Genetics	Trusted third party (TTP)	Competent clinician	Treating physician
Step 1	Submit finding	Receive finding; check sample identity; Request assessment of finding;				
Step 2			Assess finding; report to biobank;			
Step 3		Transmit pseudonym to TTP;		Resolve pseudonym; identify donor; identify competent clinic^a^);		
Step 4					Check informed consent; check medical record; decision to return the finding;	
Step 5						Inform donor; plan examination;
Step 6					Establish diagnosis; report completion to TTP;	
Step 7				Archive transaction;		

^a^An external TTP passes the information to the hospital where the competent clinic/clinician is identified.

**Fig. 1:**
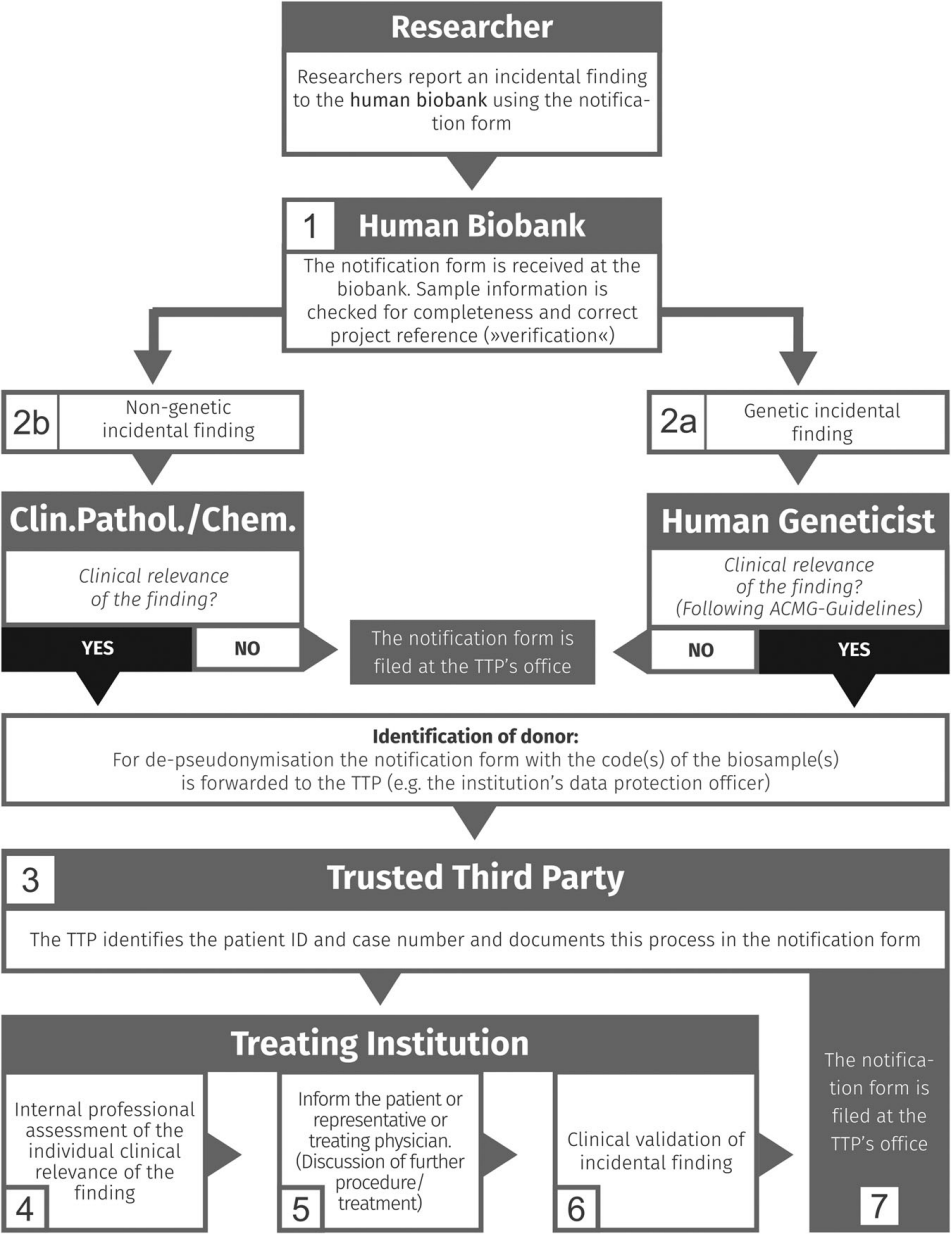
Step-by-step detailed generic workflow (including responsibilities and documentation) for a standard procedure for managing incidental research findings fed back to a hospital-based academic biobank for samples collected under a “broad consent” scheme. The seven major steps comprise (from top to bottom):—(1) verification of the samples’s provenance;—(2) a first competent assessment of the genetic (2.a) or non-genetic finding (2.b);—(3) donor de-pseudonymization in case of potential clinical relevance of the research finding, and checking the donor’s decision on receiving information on research results;—(4) re-assessment of the finding on the discretion of the competent clinician considering the donor’s individual clinical record;—(5) professional information on the potential clinical relevance of the finding to the donor by a person/treating physician(s) with sensitive communication abilities;—(6) Clinical validation of the finding;—(7) Filing of the documents in the archives of the trusted third party (TTP).

The workflow for processing a report on an incidental finding is structured as follows (see Fig. 1):

### Step 1 (Verification)

After receiving the information on an incidental finding from a researcher the biobank must ascertain that the respective biological sample was retrieved from their collection (Fig. 1, No. 1). To allow for an assessment whether the finding is still at all relevant the date when the sample has been collected is determined. This being verified, the information is handled according to its nature: For genetic findings and findings based on research methods or analytes not clinically validated, appropriate expert opinion must be obtained.

### Step 2 (Validation)

Since the finding is usually detected in a research lab which cannot provide adequate clinical validation of the results, re-assessment of the finding is imperative. The biobank should have competent experts and laboratories identified in advance whom to turn to for the assessment of genetic, pathological or analytical findings.

### (a) (genetic findings)

The information is forwarded to an appropriate institution competent for human genetics and associated or collaborating with the biobank, such as a local department of human genetics or a board certified medical geneticist. Based on experimental data and professional experience the results are evaluated by a human geneticist with regard to their validity and significance. The clinical relevance of the finding is assessed in accordance with the Guidelines of the American College of Medical Genetics and Genomics (ACMG) [2] and the German Society of Human Genetics [27]. If the finding is regarded as being valid and of clinical relevance the next step in the workflow is taken (Fig. 1, No. 2a).

### (b) (non-genetic research findings)

Depending on the finding and the analytical method used a professional with adequate expertise such as a clinical pathologist or clinical chemist receives the information for evaluation. Depending on the result of the assessment, the finding is either passed to the next step or dropped (Fig. 1, No. 2b).

### Step 3 (Identification)

Until this point the finding is only linked to the sample pseudonym and thus the identity of the donor is still unknown. The link of pseudonyms to donor identities is typically overseen by the trusted third party (TTP). In case of the ibdw this duty is assumed by the institutional data protection officer (institutional DPO) of the University Hospital Wuerzburg (Fig. 1, No. 3). The TTP (e.g. the institutional DPO) receives the pseudonym and identifies the donor and the relevant ward, clinic or department based on the time, place and context the sample has been obtained (referred to as *competent clinician*).

### Step 4 (Evaluation)

The TTP (e.g. the institutional DPO) informs a competent clinician at the institution where the patient has been treated of the donor’s identity and the finding.

### (a) (consent check)

The *competent clinician* or a person authorized by the clinician checks the medical record of the donor and the consent form to determine the donor’s decision about a potential re-contact and the “right to know” or “right not to know” (Fig. 1, No. 4a).

### (b) (case-specific re-assessment)

At the discretion of the *competent clinician* the clinical relevance of the finding may be specifically re-discussed and re-assessed by qualified medical specialists considering the donor’s medical record (Fig. 1, No. 4b). With the PII obtained, the donor’s health status can be traced and based on this (diagnosed, treated, recovered or deceased) the necessity to communicate the finding is decided.

### Step 5 (Consultation)

If the patient has consented to receive relevant findings and the significance of the finding justifies and necessitates re-contact of the donor, the respective competent clinician gets in touch with the donor to schedule an appointment for informing the donor, however without giving any details on the reason for the visit (Fig. 1, No. 5). The way in which the consultation is planned depends on the urgency of the finding and the setting. If the patient is unwilling to attend the consultation, the doctor may disclose as much in advance as needed to convince the patient of the urgency. In less urgent cases the consultation may also occur at a follow-up visit or regular checkup. The feedback procedure itself may vary, depending on the finding, involving the treating physician, the general practitioner, another trusted doctor, or geneticist, but in-person medical counseling is strongly recommended. Learning about an incidental finding can become emotionally and psychically challenging for the donor. Thus, outstanding communication abilities and interpersonal skills are imperative for the person in charge of communicating the finding.

### Step 6 (Clinical validation of research finding)

In any case, each incidental research finding requires subsequent verification and validation in a clinical routine setting with an approved or accredited method [19].

Finally, in Step 7 (Archiving) of the proposed generic workflow, the completed notification will be scanned and electronically stored for 30 years in the data archives of the TTP for reasons of transparency, quality control, and traceability according to the GDPR and according to the German Civil Code (BGB §199) (see Supplementary Fig. 1).

A feedback to the researcher reporting an incidental finding is not provided. Only the receipt of the report of the incidental finding is confirmed by the biobank.

The approach implemented by the ibdw provides a transparent workflow for transmitting incidental research findings on human biological samples and related data to the donor affected. The process fully complies with the ethical and legal framework and is designed to reliably ensure donor privacy. The process has been designed to meet the requirements of all scales of biobanks, major collection strategies of larger, hospital-based biobanks, study-specific, and broad consent-based collections of human samples.

In this process the hospital-based biobank itself acts solely as an intermediary between the researcher and the donor. The identity of the respective donor is neither disclosed to the researcher nor to the human biobank but resolved by a TTP which communicates directly with the competent clinician.

Before incidental findings are communicated to a donor the clinical significance and impact on the donor’s health is assessed. The decision on disclosure of the finding to the donor is based on generally accepted guidelines and professional experience [2, 8, 28, 29]. Yet, first and foremost the donor’s decision to exercise the “right not to know” is to be respected [20, 30, 31]. Obligations arising from statutory conditions, such as the professional law of German physicians, remain unaffected and shall nonetheless be observed [13, 18, 32].

Accordingly, in case of emergency or in case of incidental findings requiring urgent intervention, the physician may be obliged by statutory regulation to take immediate action, thus disregarding the patient’s desire for being uninformed of any incidental finding. However, incidental findings of this kind, e.g. an aneurysm of the ascending aortic arch with a high risk for rupture, are rather uncommon in biomedical research projects, while more frequent for imaging methods like MRI or CT scan [33, 34]. Yet, even under these circumstances the donor’s autonomy, health concerns and professional ethics should be carefully balanced. By this procedure all major aspects relevant to the decision on returning incidental findings and preconditions for an ethically and legally adequate procedure have been considered and incorporated appropriately.

## Discussion

With the growing importance of high-throughput analytical techniques in biomedical research which generate high-dimensional data, the chance for discovering unexpected health risks or signs for disease in human biological samples has become an issue of significant relevance. Hence, the question when, which and how research results and incidental findings may be returned to the sample donor has become a topic of many controversial debates [1, 15, 35, 36]. The position European biobanks take ranges from ruling out any return of research results to full disclosure of all observations to the sample donor [37, 38]. For example, one of the major population-based biobanks, the UK biobank, clearly stated in their ethics and governance framework document that the UK Biobank “will generally not provide health information to participants” [39]. However, with the increasing number of research projects on UK Biobank data, particularly imaging data, it was anticipated that many potentially serious incidental findings would arise, and the policy has been adjusted accordingly [14, 40].

Other biobanks either leave the decision to the donor or stipulate the return of incidental findings in the consent [8, 41–43]. Several studies indicate that a considerable share of biobank donors are decidedly interested in research findings on their samples [31, 44, 45]. Also, from the physician’s perspective the return of incidental findings appears advisable when the finding can significantly affect the donor’s wellbeing. Consequently, medical law experts, ethicists, patient advocacy groups, health professionals but also scientists demand that the return of incidental findings should be recognized as a key element to patient self-determination [1, 4, 46]. Providing sufficient and understandable information to the donor is thus imperative to enable an informed decision on the matter [47]. By involving a cancer patient advocacy group (“Kampf gegen Krebs e.V.”) the ibdw strives to incorporate patients’ concerns and desires and to align policies and procedures with patients’ needs. Considering the disturbing prospect of subsequent exhausting and burdensome diagnostic and therapeutic interventions in order to assess the individual true (validated) clinical relevance of an incidental finding calls for an empathetic and sensitive medical consultation where the donor learns about the respective finding, carefully weighing the donor’s justified interest and emotional stress. At the Medical faculty of the University of Wuerzburg the relevant skills are taught as part of the skill enhancement for medical students [48].

The biobank should strive for a timely communication of the finding to the donor [8], which may, however, not always be achievable due to privacy issues and organizational conditions.

The approach presented here for the management and communication of incidental findings was designed in consideration of applicable legal, ethical and organizational requirements for a German academic hospital-based biobank and refined by the GBA steering board in an internal discussion and improvement process. The *modus operandi* was designed to cover all relevant conditions—ethical, legal, and technical—including the collection of human biological samples on the basis of a “broad consent” scheme and providing such samples and data to researchers world-wide. By this transparent workflow, hospital-based biobanks can manage incidental research findings on human biosamples complying entirely with pertinent rules and regulations. By a sequence of checks and controls, a well balanced decision on the disclosure of the incidental finding is ascertained. After verification of the sample provenance (Step 1) an initial assessment of the genetic or non-genetic finding by an expert ensures that only clinically relevant findings are given consideration (Step 2). After the donor has been re-identified (Step 3) the donor’s decision on receiving incidental research findings is checked (Step 4.1) and a competent clinician can then reconsider the decision on returning the finding in view of the donor’s health record (Step 4.2). Finally, in due consideration of the donor’s condition and intentions, the finding, the clinical relevance and potential consequences are communicated to the donor in a professional, respectful and emphatic manner by a qualified/treating physician with excellent interpersonal and communication skills (Step 5).

By this approach the issue of incidental findings in the context of hospital-based biobanking is comprehensively addressed and may serve as a generic model for other larger hospital-based human biobanks. The implementation of this procedure does not require major changes to the informed consent. Actually, a passage on whether the patient would like to exercise the “right to know” or “the right not to know” is sufficient. For legacy samples collected under a consent which has not provided a decision on the “right to know”/”right not to know” the procedure can be applied as well if the return of an incidental finding is mandatory due to legal or statutory rules.

Since the ibdw collects samples based on a broad consent only from adults, procedures for pediatric patients are not foreseen here. Apart from considerations on possibly necessary informing [49]—or even re-consenting—of the child when coming of age, the procedure should also be applicable to pediatric patients without major changes. However, parents or a legal guardian are to be involved to take the decision on the “right to know”/”right not to know”. Before implementing this procedure for pediatric donors, the complex ethical implications of returning incidental findings [50], and balancing the rights of the child with the perspective of the parent or guardian, particularly when the onset of the disease is uncertain in the future, must be addressed.

Another exceptional case arises when the donor is deceased. While there is no guideline pertaining to the issue of returning results to the offspring of deceased in place, genetic findings indicative for a severe defect of the offspring may give reason to consider returning this observation to relatives of the deceased. Considering the difficulties in identifying and tracing descendants, an obligation to inform them appears quite disproportionate. Yet it is at all questionable whether the information would even be welcomed by the descendants. In fact, the offspring are in general not informed of their parents’ medical diagnoses even during their lifetime without explicit consent. Consequently, a corresponding regulation would have to be provided for in the biobank consent.

Owing to the rare occurrence of incidental research findings at the ibdw, the workflow was only subjected to a test for functionality. Future experience will tell if the adequacy, suitability and practicability of the procedure can be proven. In the current piloting phase the process is still paper-based. The entire process can also be implemented fully digitally, based on a computerized workflow and using a project management and ticketing system. While the ibdw already controls internal workflows in this way, the system is not yet available throughout the hospital. Provided the necessary infrastructure, as well as authentication and authorization procedures are in place, the changeover to the fully electronic process can be carried out without major difficulties.

Meanwhile, the concept and templates developed will be assessed for their applicability at other hospital-based GBA biobanks. With the experience from the test phase the process will be optimized and apparent shortcomings eliminated. Ultimately, the procedure is to be implemented more broadly and adapted to local characteristics within the German Biobank Alliance. Future pan-European dissemination can occur in collaboration with the BBMRI-ERIC infrastructure and after adaptation to the relevant national regulations.

In recent years, the concept of creating networks of many independent biobanks to collaborate, coordinate sample requests, and provide samples through a single point of access has gained increasing traction. Transparent, ethically and legally sound processes are fundamental for these networks to be able to fulfill their tasks. This is only possible if the procedures are standardized, follow established workflows and correspond to generally accepted principles.

European and National research infrastructures such as BBMRI-ERIC and GBA pursue a comprehensive harmonization of biobank procedures. This has been accomplished in many aspects, yet primarily with technical or organizational focus. The management of incidental research findings, however, remained an unresolved issue. The scheme described here provides a blueprint for a standard procedure that can be adapted and adopted by hospital-based biobanks with comparable governance structure and even extended to meet the needs of large infrastructures by adding an additional layer for the coordination of the reporting of incidental findings and complete digitization of the process.

## Supplementary information

Supplemental Figures Legend

Supplemental Figure 1

Supplemental Figure 2
