# Supplementary material for: GBA/GBN-position on the feedback of incidental findings in biobank-based research: consensus-based workflow for hospital-based biobanks
Source: Eur J Hum Genet. 2023 Feb 3;31(9):1066–72. doi: 10.1038/s41431-023-01299-8 (PMC10474025; doi:10.1038/s41431-023-01299-8)
Supplement: Supplementary file 1 — Supplemental Figures Legend [file 41431_2023_1299_MOESM1_ESM.docx]

**Supplemental Figures**

**Supplemental Figure 1:**

Step-by-step detailed generic workflow (including responsibilities and documentation) for a standard procedure for incidental research findings fed back to a hospital-based academic biobank providing human biological samples collected under a “broad consent” scheme which can be adopted by other hospital-based biobanks.

**Supplemental Figure 2:**

Template for a “notification form” for incidental findings.
