# Supplementary figures and images for: GBA/GBN-position on the feedback of incidental findings in biobank-based research: consensus-based workflow for hospital-based biobanks
Source: Eur J Hum Genet. 2023 Feb 3;31(9):1066–72. doi: 10.1038/s41431-023-01299-8 (PMC10474025; doi:10.1038/s41431-023-01299-8)

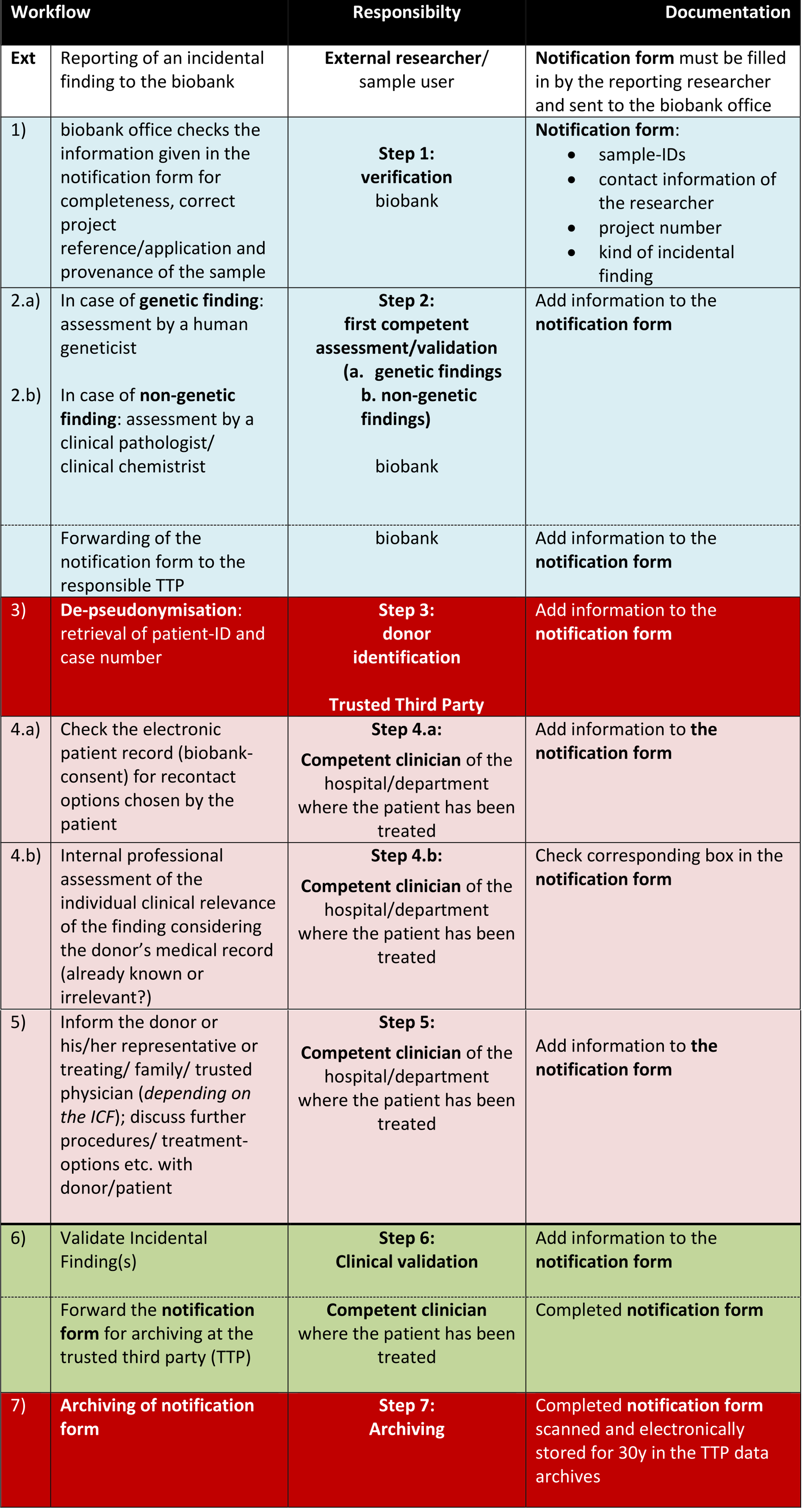

Supplement: Supplementary file 2 — Supplemental Figure 1 [file 41431_2023_1299_MOESM2_ESM.png]

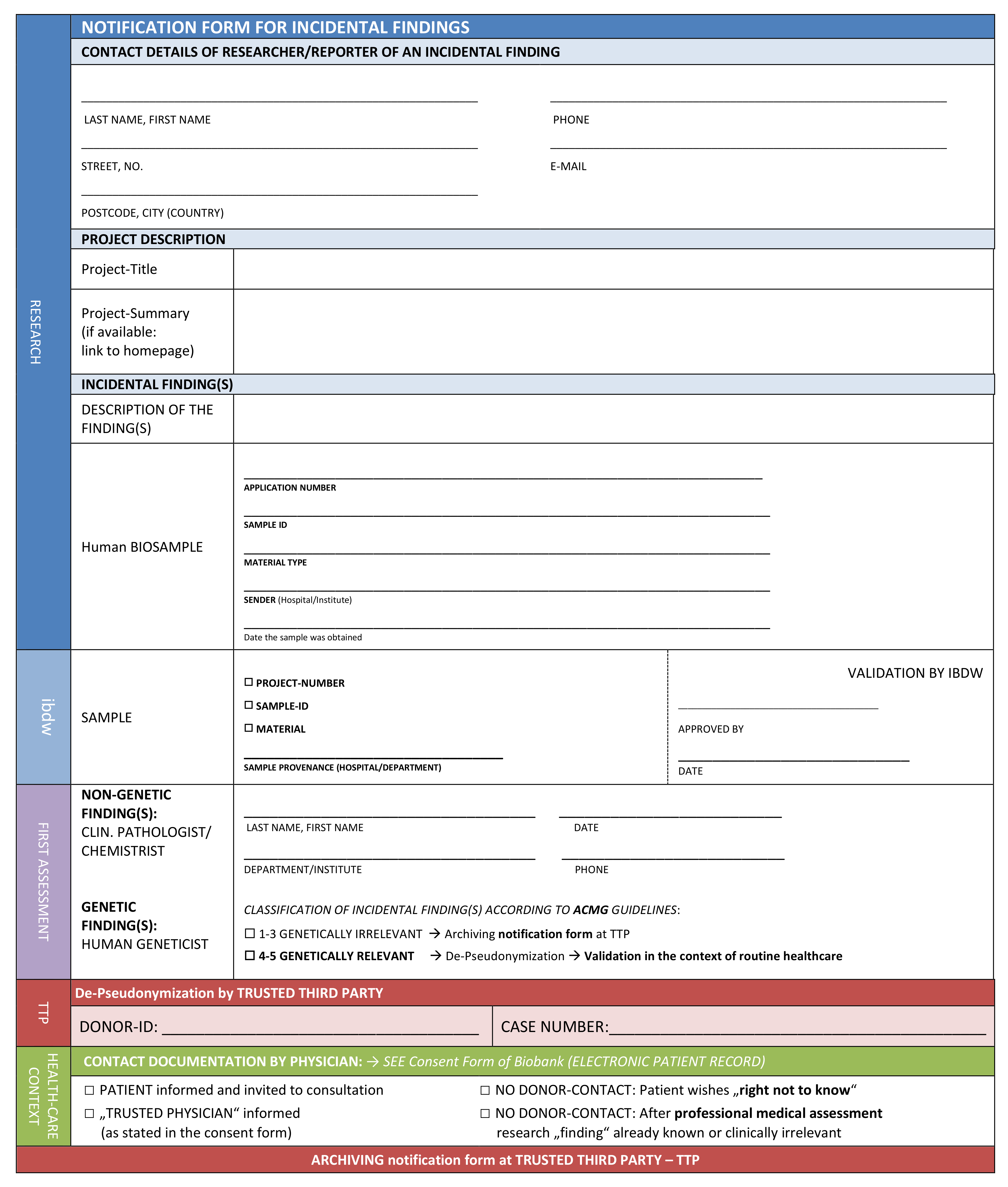

Supplement: Supplementary file 3 — Supplemental Figure 2 [file 41431_2023_1299_MOESM3_ESM.png]
